# Resection of Early Colorectal Neoplasms Using Endoscopic Submucosal Dissection: A Retrospective Multicenter Cohort Study

**DOI:** 10.3390/jcm13226989

**Published:** 2024-11-20

**Authors:** Katarzyna Winter, Przemysław Kasprzyk, Zuzanna Nowicka, Suzuki Noriko, Alberto Herreros-de-Tejada, Michał Spychalski

**Affiliations:** 1 Center of Bowel Treatment, 95-060 Brzeziny, Poland; kasprzykprzemek@gmail.com (P.K.); mspych80@gmail.com (M.S.); 2 Department of Biostatistics and Translational Medicine, Medical University of Lodz, 92-215 Lodz, Poland; zuzannow@gmail.com; 3 St Mark’s Hospital and Academic Institute, London North West Healthcare NHS Trust, Harrow HA1 3UJ, UK; norikosuzuki@nhs.net; 4 Department of Gastroenterology, Puerta de Hierro University Hospital, IDIPHISA—Instituto de Investigación Sanitaria Puerta de Hierro-Segovia de Arana, 28222 Majadahonda, Spain; alberto.herreros@salud.madrid.org; 5 Department of General and Oncological Surgery, Medical University of Lodz, 92-213 Lodz, Poland

**Keywords:** endoscopic submucosal dissection, early colorectal cancer, ESD

## Abstract

**Background:** Endoscopic submucosal dissection (ESD) is a reliable method that can replace surgery in the treatment of early colorectal cancer under certain conditions. **Aim:** The aim of the study was to analyze factors influencing the ESD procedure in early colorectal cancer, with the intention of improving its effectiveness. **Patients and Methods:** We conducted a multicenter, retrospective cohort study on 214 patients who underwent ESD procedures for early colorectal cancer from January 2016 to October 2023. **Results:** *En bloc* resection was achieved in 197 (92.1%) of ESD procedures, R0 resection in 149 (69.6%), and curative resection in 54 (40.9%). The submucosal invasion was classified as level 1 (SM1) in 96 cases (45.3%), level 2 (SM2) in 61 cases (28.8%), and level 3 (SM3) in 36 cases (17%). R0 resection was achieved more often in the rectum—92 (81.4%), compared to the right—24 (64.9%) and left colon—33 (61.1%), *p* = 0.009. In rectal tumors, R0 resection was achieved in 51 (98.1%) SM1 invasion, 27 (73%) SM2 invasion, and 13 (65%) SM3 invasion (*p* < 0.001). Lateral and vertical resection margins were positive in 12 (7.7%) and 52 (25.2%) cases, respectively. Vertical resection margins were statistically more often positive in lesions located in the right colon—11 cases (28.9%) and left colon—21 cases (38.9%), than in rectum—20 cases (17.5%); *p* = 0.010. Complications were found in 32 (15%) cases of ESD procedure—perforation in 12 cases (5.6%) and delayed bleeding in four cases (1.9%). Procedures performed in the right colon were associated with a significantly higher risk of any complications (30%) and perforations (15%) than those performed in the rectum (10.3% and 2.6%) or the left colon (13.8% and 5.2%; *p* = 0.016; *p* = 0.015), respectively. **Conclusions:** ESD for early colon cancer is a viable strategy due to its effectiveness and low complication rate. The ESD technique performed in the rectum yields the best results; however, in the right colon, it still requires careful attention.

## 1. Introduction

The endoscopic treatment of early cancerous lesions in the gastrointestinal tract has experienced a significant breakthrough in the last decade with the introduction of endoscopic submucosal dissection (ESD) [1,2,3].

The main advantage of ESD over conventional EMR is its ability to achieve a high rate of *en bloc* resection, regardless of tumor size. This leads to a more precise histological evaluation of specimen margins and a lower recurrence rate during long-term follow-up [4,5,6,7].

Colorectal cancer (CRC) is the third most common cancer worldwide [8,9]. The implementation of bowel cancer screening programs and progress in endoscopic techniques results in a growing number of early pT1 colorectal cancers detection, diagnosed approximately in 0.2% to 2% of colorectal polyps removed endoscopically [10,11,12,13]. In a large German study involving 2821, 392 screening colonoscopies, premalignant precursor lesions were found in 19.4% and cancers in 0.9%. Of the cancers, 47.3% were detected at an early stage [14]. Early CRC is defined as the “invasion of neoplastic glandular epithelial cells through the muscularis mucosae into the submucosa of the bowel wall but not beyond” [15].

Nonetheless, endoscopic resection of early colorectal cancer may present several challenges. First, it is not always possible to determine the risk of submucosal invasion before performing endoscopic resection. Therefore, choosing the best resection method can be difficult. Second, endoscopic resection can be considered curative only when the pathologically low-risk criteria are fulfilled [2,16]. Low-risk criteria for submucosal invasive cancers have been defined as submucosal invasion of less than 1000 µm, which corresponds to SM1 layer, exclusion of poor differentiation, exclusion of lymphovascular invasion, and exclusion of tumor budding [17,18,19]. When one of these criteria is not fulfilled, additional surgical resection is recommended to assess the lymph node staging [20,21]. Risks of nodal metastasis by the submucosal layer have been reported as follows: SM1 < 1%, SM2 = 6%, and SM3 = 14% [20,22,23]. Another controversial aspect is the management of neoplastic lesions in colitis. Inflammatory bowel disease is associated with an increased risk of colorectal cancer, with estimates ranging from 2 to 18% [24]. ESD can provide valuable support in the resection of even precancerous colitis-associated lesions, especially large ones with fibrosis [24,25].

The question that needs to be answered is whether endoscopic treatment offers an equal quality of oncologic therapy for early colorectal cancer in comparison to surgery [1]. Is it possible that endoscopic resection will be the method of choice in the treatment of selected patients with early colorectal cancer? And where do we have room for improvement?

In a large-scale multicenter study of long-term outcomes after endoscopic resection for submucosal invasive colorectal cancer, among the patients with low-risk features treated by endoscopic resection alone, the 5-year recurrence-free survival and recurrence rates were 98% and 0.8%, respectively [26].

So far, little data are available from the Western world regarding the efficacy of colorectal ESD, especially in submucosal carcinomas [3,16,27].

The aim of this study was to analyze factors influencing the ESD procedure in early colorectal cancer, with the intention of improving its effectiveness.

## 2. Materials and Methods

### 2.1. Study Population

We conducted multicenter, retrospective cohort study on 214 patients who underwent ESD procedures for colorectal cancer from January 2016 to October 2023. ESD procedure was conducted in the following centers: at Center of Bowel Treatment, Brzeziny (Poland), Hospital and Academic Institute; London North West Healthcare NHS Trust (UK), Puerta de Hierro University Hospital Majadahonda IDIPHISA Instituto de Investigacion Segovia Arana, Majadahonda (Spain).

Data for the study were collected through a retrospective review of medical records, a database of endoscopic reports, histopathological results, and information from hospital outpatient clinic cards. Lesions were evaluated using magnifying chromoendoscopy and were assessed according to the Paris, NICE, and Kudo’s pit pattern classifications [22,28,29,30]. Patient (e.g., age, sex, race, BMI, ASA status) and lesion characteristics (e.g., location, diameter), procedure characteristics (e.g., *en bloc* resection, procedure duration, resection speed), complications of ESD procedure (bleeding, perforations, polypectomy coagulation syndrome (PECS)), histopathologic assessment (e.g., invasion depth, grading, margins, R0 resection rate, lateral and vertical margins, vascular and lymphatic invasion), were reported. All authors had access to the study data and reviewed and approved the final manuscript.

### 2.2. ESD Procedure

The indications for ESD strictly followed Colorectal ESD/EMR Guidelines, included laterally spreading tumors (LST) of >20 mm and lesions difficult to resect with conventional EMR, i.e., lesions with non-lifting sign, or a local recurrence after earlier endoscopic treatments [2,17]. ESD was performed using the following procedures, as previously described [17,31,32].

*En bloc* resection is characterized as surgical excision of whole tumor with margin of healthy tissue. Complete histological resection (R0) refers to a surgical excision verified by the histopathological examination of post-procedure samples regarding the healthy margins. Incomplete histological resection is defined as a failure to achieve neoplasia-negative margins (R1) or when pathologist (Rx) cannot adequately evaluate margins.

For sessile tumors, a staging system adapted from the three levels of Kikuchi’s system through the classification of Paris is used to classify the depth of submucosal invasion as SM1 (<1000 μm), SM2 (1000–2000 μm) or SM3 (>2000 μm) [22,33,34].

Curative ESD procedure or success rate was defined when all of the following criteria were met: (1) resected specimen with negative lateral and deep margins of cancer cells, (2) depth of submucosal invasion < 1000 μm below the muscularis mucosae, (3) absence of poorly differentiated or mucinous histology, (4) absence of lymphovascular involvement and tumor budding, and (5) without severe complication requiring additional surgical treatment.

### 2.3. ESD Complications

Complications were classified as intra-procedural (detected during the procedure) or delayed (detected after ESD). Post-procedural bleeding was categorized as mild, requiring endoscopy without blood transfusion, and severe with loss of ≥2 hemoglobin units after completion of the ESD procedure [35]. Bleeding during ESD was considered to be a complication when it was severe, leading to premature termination of endoscopic resection. Perforation was defined as an obvious endoscopic view into the perirectal space or the peritoneal cavity, detected during the procedure or as free air in the abdominal cavity on image studies or seen during emergency surgery. Perforations detected during the ESD procedure were immediately closed with a metal clip. Complications severity was classified according to Clavien-Dindo Classification of Surgical Complications [36].

### 2.4. Ethical Considerations

This study was conducted in accordance with the ethical principles of the 1975 Declaration of Helsinki, and the study protocol was approved by the Committee of Bioethics of Medical University of Lodz, Poland (RNN/191/20/KE, 14 July 2020). All enrolled patients provided informed consent for the ESD procedure.

### 2.5. Statistical Analysis

Nominal variables are presented as numbers with percentages, and continuous variables are presented as medians with 25–75%. Continuous variables were compared between two groups using the Mann–Whitney U test and between more than two groups using Kruskal–Wallis test. Tests for nominal variables were performed using chi-square test, chi-square test with Yates correction or Fisher exact test, depending on the number of patients in compared groups. All tests were two-sided. Resection speed was calculated by taking the square of tumor diameter in mm and dividing by resection time in minutes. Multivariable analysis was performed using logistic regression to identify features independently associated with the probability of R0 resection after adjusting for patient age. Odds ratios with 95% confidence intervals (95% CI) were used for presentation. *p* values < 0.05 were considered statistically significant. Statistical analysis was performed using R version 4.4.

## 3. Results

Out of 214 patients included in the study, there were 81 female (37.9%) and 133 male (62.1%). The median age was 67 years (ranging from 35 to 93 years). The majority of the patients were Caucasian—202 (95.7%). The median body mass index (BMI) [kg/m^2^] was 27, with a range of 17.1–45.1. Most patients were ASA grade II—129 (62.2%), overweight—60 (39.7%), or of normal weight—46 (30.5%). The baseline characteristics of subjects involved in the study are presented in Table 1.

Lesions were mainly located in the rectum—116 (54.2%) and the sigmoid colon—49 (22.9%). The median tumor diameter was 40 mm (range 4.0–150 mm). A total of 67 (35.3%) lesions were classified as lateral spreading granular tumors (LST-G), 106 (55.8%) as non-granular lesions (LST-NG), and 17 (8.9%) as mixed-type. Lesions were mostly Paris 0-IIa + Is—72 (33.6%) and 0-IIa + IIc—61 (28.5%). According to the NICE classification, 145 lesions (68.7%) were classified as NICE II and 66 lesions (31.3%) as NICE III. Detailed characteristics of lesions subjected to ESD treatment are presented in Table 2.

Tumors classified as NICE III were significantly smaller than tumors classified as NICE II (*p* < 0.001).

The submucosal invasion was classified as level 1 (SM1) in 96 cases (45.3%), level 2 (SM2) in 61 cases (28.8%), level 3 (SM3) in 36 cases (17%), and in 21 (9.8%) degree of submucosal invasion could not be assessed (SMx). The degree of tumor invasion was not associated with its size (*p* = 0.058). Most of the lesions were moderately differentiated adenocarcinomas (G-2)—98 cases (48.3%) and well-differentiated adenocarcinomas (G-1)—74 cases (36.4%). A detailed characterization of the histopathological results of the lesions removed in the ESD is presented in Table 3.

*En bloc* resection was achieved in 197 ESD procedures (92.1%), R0 resection in 149 (69.6%), and curative resection in 54 (40.9%). The reason for most (88.5%) non-curative resections was invasion depth >SM1. The tumor diameter was not associated with curative resection (*p* = 0.490). Positive lateral resection margins were observed in 12 cases (7.7%), and positive vertical resection margins in 52 cases (25.2%). Invasion of the blood vessels affected 18 lesions (8.8%) and lymphatic vessels 16 lesions (7.8%). Pre-ESD biopsy was performed in another Endoscopy Unit in 43 cases (20.1%). In six lesions (3.1%), a previous resection was performed, also in another department. ESD procedure time varied from 15 to 480 min (median 60 min). Resection speed ranges from 1.1 to 202.5 mm^2^/min (median 26.7 mm^2^/min).

**Figure 1:**
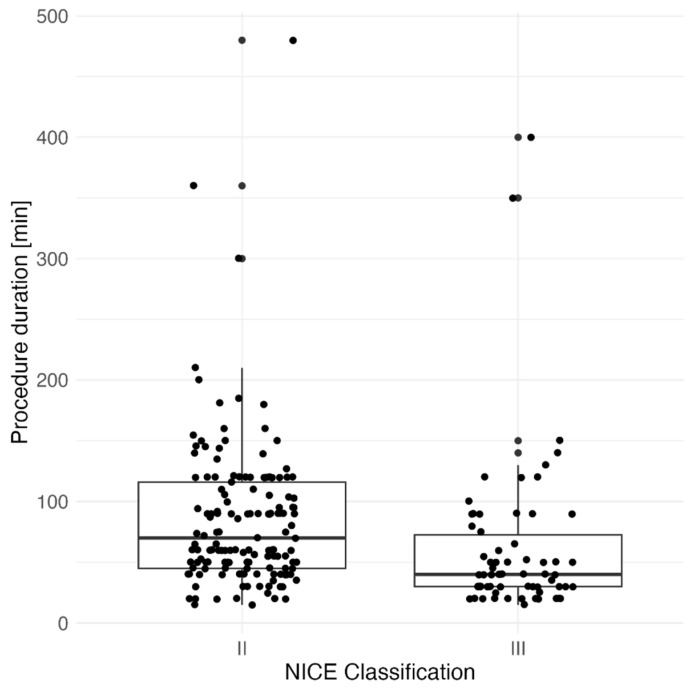
Association between the duration of tumor ESD resection [min] and NICE classification.

**Figure 2:**
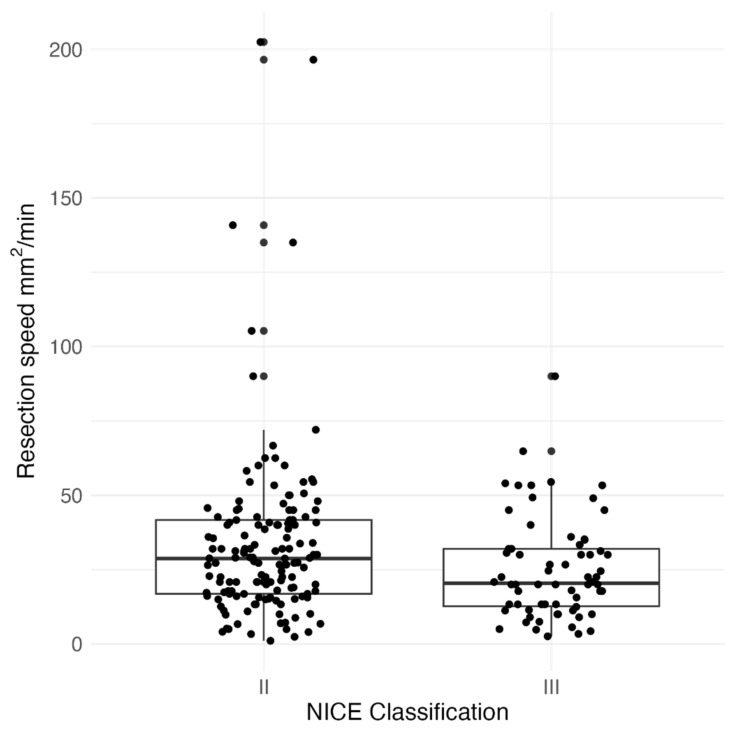
Association of tumor resection speed of ESD technique [mm^2^/min] on the tumor NICE classification.

We analyzed the factors affecting the ESD procedure with a view to improving its effectiveness. We have investigated the factors affecting the procedure’s duration and speed. The NICE classification of the lesion (II vs. III) was significantly associated with procedure duration (*p* < 0.001), Figure 1 and resection speed (*p* = 0.019), Figure 2.

**Figure 3:**
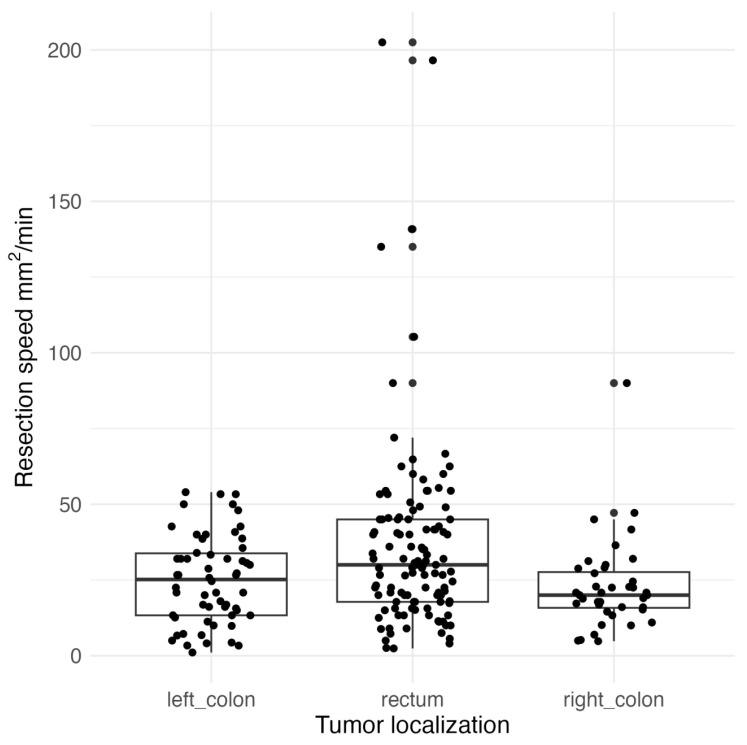
Association between ESD resection speed [mm^2^/min] and tumor location.

NICE III polyps were resected faster, and the time of the ESD procedure was shorter, probably due to the statistical difference in polyp size (*p* < 0.001). In addition, 45/66 (69.2%) of the NICE III lesions were located in the rectum. The duration of the procedure was not dependent on the location of the lesion undergoing ESD (*p* = 0.094). However, the resection speed was significantly dependent on the location of the lesion (*p* = 0.001). Resection in the rectum was significantly faster than resection in both the right (*p* = 0.005) and the left colon (*p* = 0.04), Figure 3.

There were no significant differences between the diameter of the tumor and the duration of the procedure in different parts of the colon. Patient gender (*p* = 0.585), age (*p* = 0.257), BMI (*p* = 0.276), previous resection attempt (*p* = 0.321), blood vessel invasion (*p* = 0.360), and positive vertical resection margins (*p* = 0.186) were not associated with procedure duration. Similarly, in terms of procedure speed, patient gender (*p* = 0.366), age (*p* = 0.729), BMI (*p* = 0.979), previous resection attempt (*p* = 0.438), positive vertical resection margins (*p* = 0.134) and blood vessel invasion (*p* = 0.271) were not associated.

**Figure 4:**
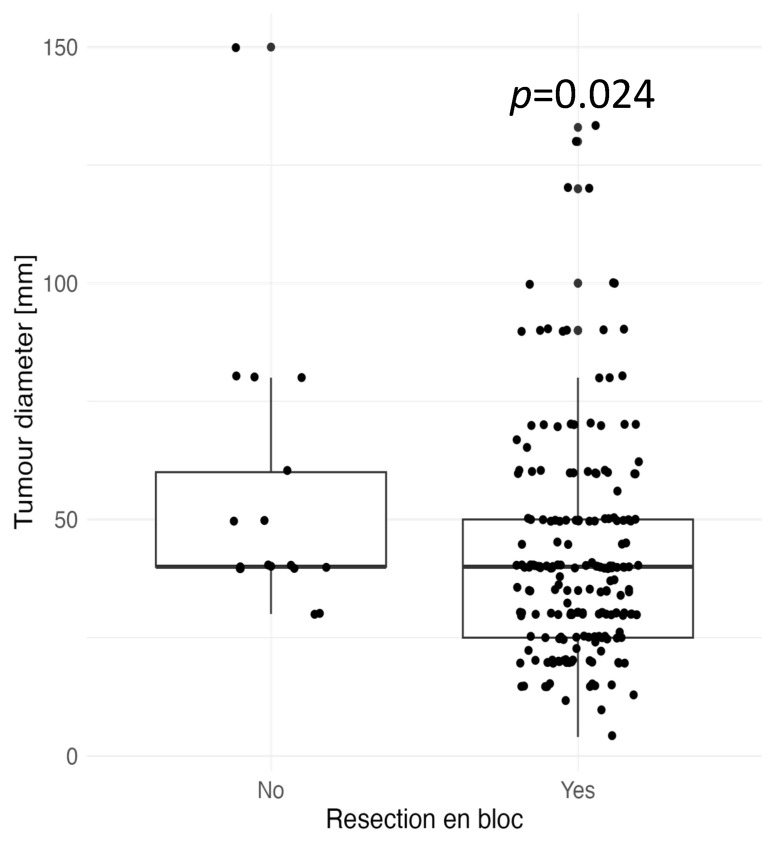
Association between *en bloc* resection and tumor diameter [mm].

We also investigated possible variables affecting failure to achieve *en bloc* resection and R0 resection, as well as factors that increase the risk of complications. *En bloc* resection was achieved in 111 tumors (95.7%) located in the rectum, 35 tumors (87.5%) located in the right colon, and 51 tumors (87.9%) located in the left colon. *En bloc* resection was statistically more frequently achieved for smaller tumors (*p* = 0.024), Figure 4.

Statistically, R0 resection was achieved more often in the rectum, 92 (81.4%), compared to the right, 24 (64.9%), and left colon, 33 (61.1%), *p* = 0.009. Resection could not be assessed (Rx) for three patients with tumors located in the rectum, three in the right colon, and four in the left colon. NICE polyp classification was not associated with R0 resection in the entire patient group (*p* = 0.731). R0 resection was not dependent on tumor size in the whole group (*p* = 0.979). However, R0 resection was achieved in 98% of tumors less than 37 mm in size. Analyzing only rectal lesions, R0 resection was achieved for 51 (98.1%) of tumors with SM1 invasion, 27 (73%) of tumors with SM2 invasion, and 13 (65%) of tumors with SM3 invasion (*p* < 0.001). NICE grade had no effect on achieving R0 resection in the rectum and tumor diameter (*p* = 0.596). In multivariable analysis corrected for patient age, tumor localization was confirmed to be significantly associated with R0 resection probability (Table 4).

More frequently, lateral resection margins were positive for lesions located in the right colon—four (16.7%) and located in the left colon—four (10.8%) than those located in the rectum—four (4.3%), however, the difference was not significant (*p* = 0.071). When analyzing vertical resection margins, the margins were statistically more often positive in lesions located in the right colon—11 cases (28.9%) and those located in the left colon—21 cases (38.9%) than those located in the rectum—20 cases (17.5%), (*p* = 0.010). Positive vertical resection margins were not associated with procedure duration (*p* = 0.186). The opposite results were obtained for lateral margins, which were significantly dependent on the duration of the ESD procedure (*p* = 0.011). Tumor size did not affect positive lateral (*p* = 0.494) and vertical (*p* = 0.804) margins. The degree of tumor invasion was significantly associated with positive vertical resection margins (*p* < 0.001) but not with lateral resection margins (*p* = 0.071). Positive vertical resection margins were observed in seven (7.5%) SM1 tumors, twenty-one (34.5%) SM2 tumors, and fifteen (44.1%) SM3 tumors.

We also decided to analyze the risk factors of vascular invasion. Blood vessel invasion was present in 7.5% of polyps classified as SM1, 11.5% of those classified as SM2, and 11.8% of those classified as SM3 (*p* = 0.538). Tumor size and location were not associated with blood vessel invasion (*p* = 0.728 and *p* = 0.945, respectively).

Complications related to the ESD procedure were observed in 32 (15%) cases. Intra-procedural complications were observed in 25 cases—perforation in 12 (5.6%) and bleeding in 13 cases (6.1%). All complications that occurred have been manageable through endoscopy. In all cases, endoclips were used during ESD to close dehiscences in muscle fibers, and further surgical intervention was not required. Post-procedural complications were identified in eight cases—post ESD electrocoagulation syndrome (PECS) in four cases (1.9%) and delayed bleeding also in four cases (1.9%). One patient had bleeding during ESD and after the procedure. No severe bleeding that required a blood transfusion was observed. There were no delayed perforations. There were no deaths related to complications. According to the Clavien-Dindo Classification of Surgical Complications [36], we classified four patients as Grade 1 and twenty-eight patients as Grade 3a.

Procedures performed in the right colon were associated with a significantly higher risk of any complications (30%) than those performed in the rectum (10.3%) or the left colon (13.8%); *p* = 0.016. (Table 5).

Procedures performed on the right colon were associated with a significantly higher risk of perforation (15%) than those performed in the rectum (2.6%) or the left colon (5.2%); *p* = 0.015. Patient sex (*p* = 0.323), age (*p* = 0.447), race (*p* = 0.365), previous resection attempt (*p* = 1.0), and tumor diameter (*p* = 0.096) were not associated with the risk of complications. Patients with ASA category III or IV significantly more often experienced any complications (28.9%) than patients with ASA category I or II (11.9%); *p* = 0.012. Patients with ASA category III or IV significantly more often experienced bleeding (21.1%) than patients with ASA category I or II (4.7%); *p* = 0.006. Patients with previous tumor biopsy statistically more often experienced any complications (48.8%) than patients with no previous biopsy (6.4%; *p* < 0.001). Patients with previous tumor biopsy statistically more often experienced bleeding (20.9%) than patients with no previous biopsy (4.1%; *p* < 0.001). Patients who experienced any complications had significantly lower BMI (*p* = 0.028).

## 4. Discussion

Endoscopic submucosal dissection, although first described by Japanese authors in the early 21st century, has only recently gained popularity in Western countries. Advances in endoscopic diagnosis and resection methods have resulted in an increased detection of early colorectal cancer. However, ESD treatment outcomes for colorectal pT1 tumors in Europe are limited. Fleischmann C. et al. [3] published the results of the German ESD Registry in the form of a prospective, uncontrolled, multicenter study, which included 20 centers with 1000 ESD records of neoplastic lesions in the digestive tract. However, only 78 procedures in the colon and 380 in the rectum were registered. Of these, only 10 adenocarcinomas were found in the colon and 57 in the rectum. *En bloc* resection in colon was performed in 84.6% with an R0 rate of 71.8% and a curative resection rate of 67.9%. *En bloc* resection in the rectum was achieved in 90.5%, R0-resection in 80.3%, and curative resection in 77.1%. Unfortunately, the data presented by the authors are for the entire ESD group without specifying the pT1 cancer group, which does not give us any relevant information regarding the effectiveness of this procedure in early cancers. Another prospective national study from France conducted between 2010 and 2013 involving 16 centers was published in 2017 [37]. The authors analyzed 288 ESD procedures, including 52 ESDs performed in the colon and 90 in the rectum. *En bloc* and R0 resection were achieved in the colon in 80.8% and 42% and in the rectum in 86.7% and 68.2%, respectively. Unfortunately, also for this analysis, the data presented by the authors are for the entire ESD group without specifying the pT1 group. So, as in the previous report, we cannot compare the results of others with ours. We published in 2021 the analysis of 601 patients who underwent ESD procedures from 2015 to 2020, with 67 cases of pT1 cancer (11.15%) [38]. The overall *en bloc* resection was achieved in 88.02% of treated patients. The R0 resection rate was reported at a level of 86.36%. These results are much better than those from other European Centers. There is only one study from Germany on ESD technique in early colon cancer. In 2017, the authors published the results of conducting an ESD in 52 large nonpedunculated rectal polyps with submucosal invasive cancer [16]. Resection rates were *en bloc* 81.4%, R0 65.1%, and curative 30.2%. The curative resection rate improved from 13.6% to 47.6% over the study period (*p* = 0.036). The reason for 83.3% (25/30) of noncurative resections was submucosal invasion exceeding 1000 µm. A meta-analysis conducted by Fuccio et al. [39] on colorectal ESD in Asian and non-Asian countries reported excellent *en bloc* resection rates of 81% in non-Asian countries and up to 93% in Asia. Although rates of curative R0 resection differed significantly between Asian (86%) and non-Asian (72%) countries, the researchers found steady progress in ESD technology in European countries. In our study, *en bloc* resection was achieved in 197 ESD procedures (92.1%), R0 resection in 149 (69.6%), and curative resection in 54 (40.9%). Analyzing en bloc resection in various parts of the colon, the best results were achieved in the rectum—in 111 cases (95.7%), then in the right colon—in 35 (87.5%), and the left colon—in 51 (87.9%), but without statistical significance. However, for R0 resection analysis, a statistically significant difference was achieved—the highest resection rate was also obtained in the rectum—92 (81.4%), compared to the right—24 (64.9%) and left colon—33 (61.1%); *p* = 0.009. Analyzing only rectal lesions, R0 resection was achieved for 51 (98.1%) of tumors with SM1 invasion, 27 (73%) of tumors with SM2 invasion, and 13 (65%) of tumors with SM3 invasion (*p* < 0.001). In multivariable analysis, corrected for patient age, tumor localization was significantly associated with the probability of R0 resection. In our previous study of 601 patients who underwent ESD, we found comparable results; lesions located in the right colon exhibited significantly lower *en bloc* (73.95%) and R0 resection rates (71.43%) [38]. These findings suggest that even endoscopic resection of early colon cancer can achieve outcomes comparable to those for polyps of different etiologies.

In a study conducted in Germany by Probst et al. [16], biopsies were taken by the referring physician in 223 out of 302 resected rectal lesions (73.8%). In 52 cases (17.2%), the submucosal invasion cancer was diagnosed, and in 30.2% of cases, the cancer was not suspected prior to resection. In our study, in 43 cases (20.1%), a previous tumor biopsy was performed (in another medical unit), probably due to the endoscopist’s lack of certainty about the nature of the lesion. In six cases (3.1%), a previous resection had been performed, also in another department. Patients with a previous tumor biopsy were statistically more likely to experience complications (48.8%), including bleeding (20.9%), than patients without (6.4%; 4.1%, respectively; *p* < 0.001). In a study conducted by Probst et al. [16], previous biopsy significantly decreased the *en bloc* resection rate to 79.4% (177/223) compared to 93.6% (44/47) in lesions without previous biopsy (*p* = 0.002). The above results indicate that the endoscopic assessment of lesions before endoscopic resection requires improvement to avoid unnecessary biopsies before resection.

We have investigated the factors affecting the procedure’s duration and the speed of dissection. In our study, the time for the ESD procedure varied from 15 min to 480 min (median 60 min), with a median lesion diameter of 40 mm (range: 4.0–150 mm). Resection speed ranged from 1.1 to 202.5 mm^2^/min. Notably, resection in the rectum was significantly faster than in both the right colon (*p* = 0.005) and the left colon (*p* = 0.04). In our previous study, the median time of dissection was 82.7 min, with a median lesion diameter of 44.3 mm [38]. We found a gradual decrease in ESD time correlated with increasing experience in clinical practice, bringing our results closer to those observed in Asian countries. According to a single-center study in Japan, the mean ESD time was 46.4 min for 1199 lesions [40]. In contrast, results from a Western country reported a median time of 105 min, with a median lesion size of 26 mm [41].

As obtaining an R0 resection is crucial in pT1 tumors undergoing ESD resection, we analyzed the risk factors for R1 resections. In our study, lateral resection margins were significantly positively dependent on the duration of the ESD procedure (*p* = 0.011). This is probably because longer procedures might have involved more difficult lesions whose margins were difficult to assess. Similarly to our results, in an analysis conducted by Lee S. et al. [42] on 527 colorectal lesions, procedure time was longer in the lateral margin-positive group than in the R0 group (94.3 ± 75.1 vs. 54.1 ± 48.9 min; *p* < 0.001). Lateral margin positivity was associated with ESD time ≥ 120 min in the multivariate analysis. In the same study, tumors were significantly larger in the lateral margin-positive group than in the R0 group (45.7 ± 21.1 mm and 30.6 ± 15.1, respectively; *p* < 0.001). However, in our study, tumor size did not affect positive lateral (*p* = 0.494) and vertical (*p* = 0.804) margins. Similarly, in Belgium, a study by Dessain A. et al. [43], specimen size was not significantly associated with positive margin rates in colorectal ESD.

When analyzing vertical resection margins, the margins were statistically more positive in lesions located in the right colon (11 cases, 28.9%) and those located in the left colon (21 cases, 38.9%) than those located in the rectum (20 cases, 17.5%; *p* = 0.010). The degree of tumor invasion was significantly associated with positive vertical resection margins (*p* < 0.001). In a recent systemic review and meta-analysis by Gu F. et al. [44] on unsuccessful colorectal ESDs, authors reported comparable results. Factors such as lesion diameter ≥40 or 50 mm, right-side colonic location, deeper submucosal invasion, and severe fibrosis were identified as risk factors for incomplete resection. Zhang Q.W. et al. [45] analyzed the rate of positive vertical margins for 489 early colorectal cancers (CRCs) in comparison to 753 advanced adenomas. The pTis early CRCs exhibited a similar rate of positive vertical margins as advanced adenomas for *en bloc* ESD (1.82% vs. 1.02%, *p* = 0.659). Additionally, pTis carcinoma was not found to be a risk factor for positive vertical margins by *en bloc* ESD (*p* = 0.368). The *en bloc* resection achieved for pT1a carcinomas exhibited comparable results to positive vertical margins achieved through ESD for advanced adenomas (2.06% vs. 1.02%, *p* = 1.000). While pT1a invasion was identified as a risk factor for positive vertical margins in lesions with *en bloc* EMR (odds ratio = 23.90, *p* = 0.005), it was not a significant risk factor in ESD (OR = 2.96, *p* = 0.396). These findings, consistent with our previous results, suggest that the ESD technique in the endoscopic resection of early colon cancer can yield outcomes comparable to those for other types of polyps.

It is already well known that the ESD technique is associated with a higher rate of complications than EMR but is also less invasive than surgery [1,7,17,29,30,46,47,48]. In our study, complications related to the ESD procedure were observed in 32 cases (15%). Intra-procedural perforations were observed in 12 patients (5.6%), while no bleeding was noted in 16 patients (7.5%). All complications were easily manageable through endoscopic intervention.

A single-center European study on ESD in colorectal lesions > 20 mm, published in 2016 [49], reported a perforation rate of approximately 9% to 10%. However, the rate of perforations leading to emergency surgery was low, at around 1%. In a Japanese study by Terasaki M. et al. [50], delayed bleeding occurred in 25 (6.6%) of 377 colorectal neoplasms post-ESD, with rectal location identified as a significant independent factor for its occurrence. In contrast, our study found that the risk of bleeding was not associated with tumor location (*p* = 0.939).

Furthermore, patients with ASA category III or IV experienced complications significantly more often (28.9%) and bleeding (21.1%) than patients with ASA category I or II (*p* = 0.012 and *p* = 0.006, respectively). Similarly, patients with previous tumor biopsy statistically more often experienced any complications (48.8%) and bleeding (20.9%) than patients with no previous biopsy (*p* < 0.001). These complications may be attributed to poor health, comorbidities, and fibrosis resulting from previous biopsies.

A meta-analysis by Akintoye et al. [51] compared complications following colorectal ESD between Asian and Western countries, revealing that bleeding and perforation rates in Asian countries were 2.19% and 3.98%, respectively, while in Western countries, the rates were notably higher at 7.2% and 7.8%. This disparity underscores the need for further research to identify and mitigate the risk factors associated with complications in different populations.

According to European ESGE Guidelines and Japanese JSCCR Guidelines, endoscopic resection in early pT1 CRC can be considered curative only when pathological low-risk criteria are fulfilled [2,20]. In cases of non-curative ESD or R1 resection, additional salvage surgical resection is recommended to assess lymph node staging. However, it is important to note that non-curative ESD does not negatively affect the patient’s long-term outcomes [52].

Yamashita et al. [52] reported the oncological results of patients who underwent surgical bowel resection due to ineffective ESD in pT1 colorectal cancer. The 5-year overall survival rate and 5-year disease-free survival rates were comparable to those of patients who underwent direct surgical intervention. Despite the increased risk of lymph node metastasis (LNM) in patients with submucosal invasion > SM1 layer, LNM has not been detected in more than 85% of patients with early pT1 CRC who undergo surgery after therapeutic endoscopy, even when assessed for the risk of LNM [53].

These findings underscore the importance of careful patient selection and the fulfillment of low-risk criteria for effective management of early pT1 CRC. Furthermore, they highlight the potential for successful outcomes following salvage surgical resections, suggesting that the initial treatment approach, even if deemed non-curative, does not significantly compromise long-term prognoses. Ongoing research is essential to refine criteria for optimal intervention strategies and to improve patient outcomes in this challenging cohort.

Although direct comparative studies between ESD and surgery are lacking, a retrospective comparison of both methods for early colorectal cancer indicated a better quality of life for patients after ESD [1,46]. According to the study by Gameldin et al. [47], who compared the ESD and laparoscopic resection, the risk of complications was significantly higher in the surgical group—21% compared to 15% in the ESD group.

Furthermore, Antonelli G. et al. [48] conducted a meta-analysis on recurrence and cancer-specific mortality following endoscopic resection of low- and high-risk pT1 CRCs managed non-surgically. Their pooled estimates of adverse events suggested a nonsurgical approach may be favorable for low-risk lesions. In a large-scale multicenter study of long-term outcomes after endoscopic resection for submucosal invasive CRC, patients with low-risk features who were treated solely by endoscopic resection demonstrated impressive results, with a 5-year recurrence-free survival rate of 98% and a recurrence rate of just 0.8% [24].

These findings highlight the potential benefits of ESD not only in terms of safety and reduced complications but also in promoting a better quality of life for patients. They further reinforce the argument for prioritizing endoscopic approaches for suitable candidates with low-risk pT1 CRC, suggesting that careful patient selection and adherence to established guidelines can yield excellent long-term outcomes without the need for more invasive surgical interventions.

There is also an interesting retrospective analysis conducted by Spadaccini M. et al. [54] on 207 non-curative ESDs for submucosal invasive cancer. In 65.2% of cases, complete resection was not achieved (R1). Among the 207 cases, 60.9% (*n* = 126) underwent surgical treatment, while 39.1% (*n* = 81) were monitored through endoscopy. Notably, patients in the follow-up group experienced significantly higher overall mortality (HR = 3.95) due to non-CRC causes (*n* = 9, mean survival after ESD 23.7 ± 13.7 months). Throughout the follow-up period, tumor recurrence and disease-specific survival rates did not differ significantly between the groups (median follow-up: 30 months; range: 6–105). Therefore, in high-risk patients, a strategy of follow-up alone may be a reasonable choice. However, endoscopic treatment is likely to become the preferred method for selected patients with pT1 colorectal cancer. ESD should be offered for lesions at risk of submucosal invasion to achieve R0 resection, optimize histopathological diagnosis, and minimize recurrence risk. Nevertheless, to improve curative resection rates, further advancements in pretherapeutic diagnostic techniques are essential. Future research should focus on refining patient selection criteria, enhancing imaging modalities, and developing protocols for accurate assessment of invasion depth, thereby optimizing treatment strategies for early colorectal cancer and solidifying ESD as a standard approach for managing early-stage lesions.

Our present study has certain limitations. Firstly, it is a retrospective analysis with missing data, which results in small sample sizes that may not provide sufficient statistical power to discern slight differences between groups. Secondly, the absence of budding in histological assessment represents a significant risk factor for lymph node metastases. Thirdly, the lack of information regarding surgical interventions, adjuvant therapies, and follow-up was a limitation, but these factors were beyond the scope of our current analysis.

Most importantly, we aim to highlight that in Western Europe, within standard clinical settings, satisfactory outcomes can be achieved in the endoscopic treatment of early colorectal cancer. These findings support the growing body of evidence suggesting that endoscopic techniques, such as ESD, are viable alternatives to traditional surgical approaches, particularly for patients with low-risk features. Further studies with larger, well-defined cohorts are warranted to validate these results and refine treatment protocols, improving patient care in this field.

## 5. Conclusions

The ESD technique is poised to become a popular and standardized treatment modality, capable of providing radical cures for certain pT1 cancers while reducing the incidence of unnecessary additional surgeries. Colorectal ESD has demonstrated effectiveness and relative safety for pT1a lesions, particularly those located in the rectum. However, attention must be given to the application of ESD in the right colon and in patients with compromised health. Furthermore, advancements in pretherapeutic diagnostic techniques are essential to enhance curative resection rates. Overall, ESD represents a promising approach in the management of early colorectal cancer, warranting further investigation and refinement.

## Figures and Tables

**Table 1:** Baseline characteristics of the study group.

Age [years]		67 (35–93) *
Sex	Female	81 (37.9%)
	Male	133 (62.1%)
Race	Caucasian	202 (95.5%)
	Asian	9 (4.3%)
	Missing data	3
ASA status **	I	40 (19.3%)
	II	129 (62.2%)
	III	36 (17.4%)
	IV	2 (1%)
BMI [kg/m^2^] ***		27 (17.1–45.1) *
	<18.5	2 (1.3%)
	18.5–24.99	46 (30.5%)
	25.0–29.9	60 (39.7%)
	≥30	43 (28.5%)
	Missing data	63

* Median (min–max). ** American Society of Anaesthesiology Scale. *** Body mass Index.

**Table 2:** Characteristics of the lesion subjected to ESD treatment.

Localization	Cecum	10 (4.7%)
	Ascending colon	15 (7%)
	Transverse colon	15 (7%)
	Descending colon	9 (4.2%)
	Sigmoid colon	49 (22.9%)
	Rectum	116 (54.2%)
Gross morphology	LST-G	67(35.3%)
	LST-NG	106 (55.8%)
	LST-mixed	17 (8.9%)
	Missing data	24
Paris Classification	0-Is	43 (20.1%)
	0-IIa	33 (15.4%)
	0-IIb	2 (0.9%)
	0-IIc	2 (0.9%)
	0-IIa + c	61 (28.5%)
	0-IIa + Is	72 (33.6%)
	0-Is + IIc	1 (0.5%)
Kudo’s Pit pattern Classification	IIIs	55 (26.7%)
	III_L_	18 (8.7%)
	IV	52 (25.1%)
	V_I_	67 (32.4%)
	V_N_	15 (7.2%)
NICE Classification **	I	0 (0%)
	II	145 (68.7%)
	III	66 (31.3%)
	Missing data	3
The diameter of the tumor [mm]		40 (4–150) *
Duration time of the procedure [min]		60 (15–480) *
Resection speed [mm^2^/min]		26.7 (1.1–202.5)
Previous tumor biopsy		43 (20.1%)

* Median (min–max). LST-G—Laterally spreading tumors granular type. LST-NG—Laterally spreading tumors nongranular type. ** NBI International Colorectal Endoscopic Classification.

**Table 3:** The characteristics of the histopathological results of the lesions.

Degree of tumor invasion	SM1	96 (45.3%)
	SM2	61 (28.8%)
	SM3	36 (17%)
	SMx	21 (9.8%)
Tumor grading	G1	74 (36.4%)
	G2	98 (48.3%)
	G3	14 (6.9%)
	Gx	17 (8.4%)
	Missing data	11
Resection *en bloc* achieved	Yes	197 (92.1%)
	No	17 (7.9%)
Resection R0 achieved	Yes	149 (69.6%)
	No	55 (25.7%)
	Rx	10 (4.7%)
Positive vertical resection margin	Yes	52 (25.2%)
	No	154 (74.8%)
	Missing data	8
Positive lateral resection margin	Yes	12 (7.7%)
	No	143 (92.3%)
	Missing data	59
Vascular invasion	Yes	18 (8.8%)
	No	187 (91.2%)
	Missing data	9

**Table 4:** Multivariable analysis of the R0 resections.

	OR (95% CI)	*p*
Intercept	0.21 (0.02–2.09)	0.184
Tumor diameter [mm]	0.998 (0.984–1.013)	0.763
Localization (rectum vs. left colon)	3.05 (1.43–6.63)	0.004
Localization (right colon vs. left colon)	0.95 (0.39–2.36)	0.919
NICE classification of the lesion (III vs. II)	0.76 (0.36–1.63)	0.478
Age [years]	1.03 (0.999–1.07)	0.056

**Table 5:** Complications of ESD depending on the location of the tumor.

	Rectum	Right Colon	Left Colon
Any complications	12 (10.3%)	12 (30%)	8 (13.8%)
Intra-procedural complications	10 (8.6%)	8 (20%)	7 (12.1%)
Any bleeding	8 (6.9%)	3 (7.5%)	5 (8.6%)
Perforations	4 (5.2%)	5 (3.4%)	3 (5.2%)

## Data Availability

The original contributions presented in the study are included in the article, further inquiries can be directed to the corresponding author.
